# Liver Resection for Non-Colorectal, Non-Carcinoid, Non-Sarcoma Metastases: A Multicenter Study

**DOI:** 10.1371/journal.pone.0120569

**Published:** 2015-03-26

**Authors:** Guillaume Martel, Jeff Hawel, Janelle Rekman, Kristopher P. Croome, Kimberly Bertens, Fady K. Balaa, Roberto Hernandez-Alejandro

**Affiliations:** 1 Ottawa Hospital Research Institute and Liver and Pancreas Surgery Unit, Division of General Surgery, University of Ottawa, Ottawa, ON, Canada; 2 Hepatobiliary Surgery, Division of General Surgery, London Health Sciences Centre, University of Western Ontario, London, ON, Canada; National Yang-Ming University, TAIWAN

## Abstract

**Background:**

The role of liver resection for non-colorectal, non-neuroendocrine, non-sarcoma (NCNNNS) metastases is ill-defined. This study aimed to examine the oncologic outcomes of liver resection in such patients.

**Methods:**

A retrospective analysis of liver resection for NCNNNS metastases was performed at two large centers. Liver resection was offered selectively in patients with stable disease. Oncologic outcomes were examined using the Kaplan-Meier method.

**Results:**

Fifty-two patients underwent liver resection for NCNNNS metastases. Overall 5-year survival was 58%. Five-year survival was 85% for breast metastases, 66% for ocular melanoma, 83% for other melanomas, 50% for gastro-esophageal metastases, and 0% for renal cell carcinoma metastases. A contemporary colorectal liver metastasis cohort had a survival of 63% (p=0.89).

**Conclusions:**

Liver resection is an effective option in the management of selected patients with NCNNNS metastases which have been deemed stable. Five-year survival rates were comparable to that of a contemporary cohort of patients with colorectal liver metastases in carefully selected patients. Further, larger studies are required to help identify potential prognostic variables and aid in decision-making in this heterogeneous population.

## Introduction

The beneficial role of liver metastasectomy for colorectal cancer (CRC) and neuroendocrine tumor (NET) origin is well established [1, 2]. With the evolution of surgical techniques, and the advancement of perioperative chemotherapy regimens, five-year survival rates greater than 60% are now reported for colorectal liver metastases [3]. Similarly, patients with metastatic NET of the GI tract, particularly midgut carcinoids, have also been shown to benefit from liver resection, both in terms of survival benefit and for the palliation of disease-related symptomatology [4, 5]. More recently, liver resection has also become a realistic treatment option for metastatic sarcomatous lesions, particularly gastrointestinal stromal tumors (GIST) in the context of effective tyrosine kinase inhibitors. Recent selected studies of sarcomas/GIST report a 5-year survival rate of 49% [6] and 3-year survival rate of 68% [7] after metastasectomy.

The role of liver resection for non-colorectal, non-neuroendocrine, non-sarcoma (NCNNNS) metastases remains ill-defined in the current literature. Indeed, the likelihood of finding favorable isolated liver metastases from NCNNNS tumors is low, perhaps owing to distant hematogenous seeding [8]. Despite this, recent studies have shown that such resections are being carried out with increasing frequency with adequate operative outcomes [9–11]. Five-year survival rates following liver resection for non-traditional metastases have varied widely in the literature (19–57% [5]), with the two largest series reporting 31% [11] and 36% [10], respectively.

The current literature is plagued with problems including the heterogeneity of primary tumor types, unknown primaries, small sample sizes in individual studies, as well as the lack of prospective studies. Of particular concern with the current literature is the frequent inclusion of NET and sarcomatous lesions, which have already been shown to benefit from hepatic resection of metastases. Relatively few studies have specifically examined the oncologic outcomes of liver resection for NCNNNS metastases, such that additional data are required to help guide clinical decisions in this population of patients not traditionally considered for liver metastasectomy. The objective of this work was thus to assess the efficacy and long-term outcomes of patients undergoing liver resection for NCNNNS metastases.

## Patients and Methods

A retrospective analysis at two tertiary hepatobiliary centers was performed to identify all patients who underwent liver resection for NCNNNS metastases between 1990 and 2012. Ethics approval for this study was sought and obtained from the Ottawa Health Science Network Research Ethics Board (2011698-01H) and the Lawson Health Research Institute Research Ethics Board (18996E). Clinical data pertaining to individual patients were obtained from medical charts and surgeon records. As allowed by both research ethics board, consent was not obtained from patients prior to the review of their medical records. However, all patient information was anonymized and de-identified.

All patients with true NCNNNS liver metastases were included. Patients with extrahepatic disease were evaluated individually for inclusion or exclusion by a multi-disciplinary tumor board consisting of hepatobiliary surgeons, medical and radiation oncologists, and radiologists. Those with metastases from CRC, NET, or sarcoma/GIST primaries were excluded. Primary tumors of the biliary tree or the liver itself were also excluded, as were tumors with direct invasion into the liver.

Patients were considered for liver resection on a case-by-case basis. Chemotherapy was not routinely administered prior to liver surgery, except for those with metastatic breast cancer or those undergoing liver resection at the same time as the primary tumor resection. Liver resection was only considered in patients who were functionally fit for major surgery, and for whom it was technically feasible to achieve an R0 resection margin while maintaining an adequate future liver remnant (FLR). Volumetric assessment of the FLR was not routinely performed. Patients with cirrhosis or portal hypertension were not considered for surgery. All patients had normal preoperative biochemical liver function tests. No other test was used to assess preoperative liver function.

Data extracted and analyzed included patient demographics, timing of presentation, tumor characteristics, type of resection, time interval to appearance and location of hepatic metastases, extent of hepatic resection, size of largest metastatic deposit, number of liver lesions, any prior history of metastatic disease, complications, chemotherapy treatment details, disease-free interval, long-term outcome, site and treatment of recurrences, and follow-up duration.

Liver metastases diagnosed within 6 months of the primary tumor were defined as synchronous, whereas metachronous presentation was defined as absence of liver metastases during the initial metastatic work-up. In the metachronous group, the time interval between the appearance of metastases from the date of diagnosis of the primary tumor was calculated. Major hepatic resection was defined as resection of three or more hepatic segments, while R0 (curative resection) was defined as total removal of all apparent liver tumor(s) with negative microscopic margins on pathology. Any histologic infiltration of the specimen margin with tumor cells was described as an R1 resection. Postoperative mortality and morbidity were tracked within 90 days of liver resection. Overall survival (OS) and disease free survival (DFS) was recorded from the date of hepatectomy until the date of recurrence or death. Survivors were censored at the date of last follow up, or death due to alternate causes in the case of disease-free survival.

The best results for hepatic metastasectomy are in CRC primaries, particularly in the modern era of chemotherapy. A contemporary cohort of patients undergoing liver resection for colorectal liver metastases (CRLM) was thus selected for comparison from our prospectively maintained database. CRLM patients from the last 10 years, between January 2002 and January 2012 were identified, and a survival comparison with the NCNNNS population was performed. In this cohort, preoperative liver function was examined in the same way as for the NCNNNS cohort. Neoadjuvant combination chemotherapy was routinely administered prior to liver resection for CRLM.

Summary statistics were generated as proportions for categorical variables and as medians with interquartile ranges for continuous variables. Survival outcomes were analyzed using the Kaplan-Meier and actuarial life tables methods. Dichotomous outcomes were compared using Fisher’s exact test, while time-to-event data were compared using the log-rank test. All statistical analyses were performed using SAS version 9.2 (SAS Institute, Inc., Carry, NC).

## Results

Between 1990 and 2012, 52 patients underwent liver resection for metastatic NCNNNS tumors at our two centers. The primary tumor sites for this cohort are presented in table 1. The two most common primary tumors were melanoma and breast carcinoma (21% each).

**Table 1:** Sites of primary tumors for those that underwent liver resection for NCNNNS metastases.

Site	Number of patients (n = 52)
Breast	11 (21%)
Melanoma	11 (21%)
Ocular	5 (9.6%)
Cutaneous	5 (9.6%)
Small bowel	1 (1.9%)
Esophageal/Gastric	7 (13%)
Renal	4 (7.5%)
Adrenal	3 (5.7%)
Ovarian	3 (5.7%)
Pancreatic	3 (5.7%)
Duodenum	2 (3.8%)
Testicular *	2 (3.8%)
Thyroid **	2 (3.8%)
Ampullary	1 (1.9%)
Endometrium	1 (1.9%)
Lung	1 (1.9%)
Unknown	1 (1.9%)

Abbreviations—NCNNNS: non-colorectal, non-neuroendocrine, non-sarcoma metastasis.

* The histology of the 2 testicular cancers was non-seminomatous & yolk sac.

** The histology of the 2 thyroid cancers was medullary carcinoma.

The clinical characteristics of included patients are summarized in Table 2. Synchronous metastases were present in 10 (19%) patients. The median disease-free time interval between treatment of the primary tumor and liver resection was 20 months (0–267) (median 0 months for synchronous lesions, 34 months for metachronous lesions). Bilobar disease was found in 13 patients (25%). Five patients (9.6%) had had previous surgical treatment for other systemic metastases (ovarian carcinoma n = 3, ocular melanoma n = 2). In total, 17% of patients had resectable extrahepatic disease at the time of presentation for consideration of hepatic resection. Resectable extrahepatic sites in individual patients included lung, axilla, tumor thrombus within the vena cava, omentum, prior metastases, adenopathy of porta hepatis, and questionable lumbar spine metastasis. Two patients had resectable retroperitoneal adenopathy. Only 20% of patients had neoadjuvant chemotherapy prior to liver resection, whereas 63% had adjuvant therapy.

**Table 2:** Demographics & Metastatic Characteristics.

Characteristics	Number of patients (n = 52) / Median (range)
Gender (male/female)	17 (33%) / 35 (67%)
Age, years	58 (35–76)
Number of metastases	
1	33 (63%)
2	8 (15%)
3	5 (9.6%)
4+	6 (12%)
Liver involvement	
Unilobar	39 (75%)
Bilobar	13 (25%)
Presentation	
Synchronous	10 (19%)
Metachronous	42 (81%)
Resectable extrahepatic lesions	9 (17%)
Chemotherapy*	
Neo-adjuvant	7 (20%)
Adjuvant	22 (63%)
None	5 (14%)
Chemoembolization	1 (2.9%)

* Data on chemotherapy is missing for 17 patients.

The different types of surgical resections are shown in Table 3. Major liver resection was performed in 21 (40%) patients. An R0 resection was achieved in 93% of our patients, and 7% were found to have R1 margins at pathology. There were no patients with macroscopically positive margins (R2).

**Table 3:** Operations carried out and perioperative outcomes.

Variable	Number of patients (n = 52)
*Operations*	
Right hepatectomy	11 (21%)
Extended right hepatectomy	3 (5.8%)
Left hepatectomy	4 (7.7%)
Extended left hepatectomy	3 (5.8%)
Left lateral sectionectomy	11 (21%)
Segmental resection	9 (17%)
Wedge resection	10 (19%)
Other	1 (1.9%)
*Postoperative complications*	
Intraabdominal abscess	5 (9.6%)
Major hemorrhage	4 (7.7%)
Bile leak	2 (3.8%)
90-day Mortality	0

Major complications, defined as Clavien-Dindo class 3 and higher, related to hepatic resection occurred in 11 patients (21%). Significant hemorrhage occurred in 4 patients (7.7%), while 2 patients (3.8%) developed bile leaks that were managed with a combination of biliary stenting and percutaneous drainage. Five patients (9.6%) developed intraabdominal abscesses requiring percutaneous drainage under radiologic guidance. There were no 90-day mortalities.

The median overall follow-up for the cohort was 54.7 months (4.5 years). Overall 5-year survival was 58%. In total, 12/52 (23%) were lost to follow-up prior to 5 years and were censored. Among those who were not censored prior to 5 years, there were 22/40 (55%) actual 5-year survivors. Among those who were not censored prior to 10 years, there were 4/35 (11%) actual 10-year survivors. Five-year OS was 100% for adrenal carcinoma, 85% for breast carcinoma, 66% for ocular melanoma, 83% for other melanomas, 50% for gastroesophageal carcinomas, 0% for renal cell carcinoma, and 46% for others. Among the 22 patients who actually survived to 5 years, 59% had breast cancer or melanoma metastases. Detailed survival and recurrence data are presented in table 4.

**Table 4:** Summary of oncologic outcomes.

Outcome	Breast (n = 11)	Melanoma (n = 11)	Esophageal/ gastric (n = 7)	Renal (n = 4)
Median DFI, mo	6	78	8	36
Median follow-up, mo	61.2	73.5	37.5	29.8
Median OS, mo	64.3	100.4	60.6	21.2
1-year OS	89%	90%	100%	75%
3-year OS	89%	81%	60%	50%
5-year OS	89%	81%	60%	0%
Median DFS, mo	19	10.5	21.6	21
Recurrence*	8 (73%)	7 (64%)	5 (71%)	2 (50%)
	**Adrenal (n = 3)**	**Pancreas (n = 3)**	**Ovarian (n = 3)**	**Others (n = 10)**
Median DFI, mo	48	28	27	2
Median follow-up, mo	57.4	54.4	62.7	34.5
Median OS, mo	64.8	54.4	89.9	21.2
1-year OS	100%	100%	100%	88%
3-year OS	100%	50%	100%	63%
5-year OS	100%	50%	100%	38%
Median DFS, mo	34.2	4	8	4.5
Recurrence*	2 (67%)	2 (67%)	2 (67%)	8 (80%)
	**CRLM (n = 185)**			
Median DFI, mo	NA			
Median follow-up, mo	35.3			
Median OS, mo	63.5			
1-year OS	94.4%			
3-year OS	75.9%			
5-year OS	60.6%			
Median DFS, mo	36.6			
Recurrence*	73 (39%)			

Abbreviations—DFI: disease-free interval (primary to metastasis); DFS: disease-free survival (after liver resection); mo: months; OS: overall survival.

*Certain patients had both local/regional recurrence as well as distant recurrence.

**Fig 1:**
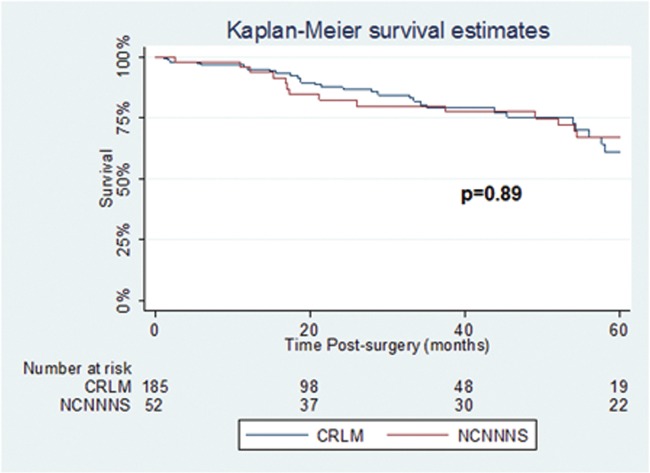
Overall survival of patients in the NCNNM cohort compared to the CRLM cohort.

Survival of the NCNNNS cohort compared to the CRLM cohort (N = 185) undergoing liver resection during the same time period can be seen in fig. 1. Mean age of the CRLM cohort was 57.7±18.9. Median follow-up for CRLM cohort was 35.3 months (2.9 years). Overall 5-year survival for the CRLM cohort was 63%. There was no statistically significant difference in survival between the 2 groups (p = 0.89).

## Discussion

Resection of liver metastases for cancer primaries other than CRC, NET, and sarcoma is a relatively new concept. However, progress in liver surgery and the success of colorectal metastasectomy have encouraged the development of surgical strategies and research to cope with patients presenting with liver metastases from other primary tumors. This study addressed the oncologic outcomes of this group at two high-volume liver surgery centers in Canada.

Patients that present with liver metastases from NCNNNS generally have a poor prognosis. Thorough evaluation and rigorous patient selection must occur before consideration of liver resection. Standard selection criteria for liver surgery, namely the life expectancy of an individual, performance status, co-morbidities, and the technical resectability of a given tumor burden must all be considered. However, in the setting of NCNNNS metastases, oncologic selection criteria must predominate, as the ability to control the systemic disease burden with chemotherapy is generally more limited. Exclusion of additional extra-hepatic metastases and loco-regional recurrence is of particular importance. At our centers, patients were typically assessed with CT and/or MRI. In addition, PET-CT scan is increasingly utilized in preoperative staging and patient selection [12]. PET-CT can be especially helpful in identifying metastatic perihepatic lymph nodes, which are a poor prognostic sign [13]. The option of staging laparoscopy as a patient selection tool has been described. In a study by D’Angelica *et al*, 20% of patients who had a staging laparoscopy were spared a laparotomy due to extent of disease [14].

Thoughtful patient selection necessitates a good understanding of the heterogeneity of the NCNNNS liver metastasis population. Distinct primary tumor biology and a low incidence make this a difficult group to study. Determination of prognostic factors has therefore been attempted in other studies [10]. Favorable prognostic indictors include genitourinary, breast, and soft tissue site primaries, a curative (R0) resection, as well as metachronous metastases [15–20]. Poor prognostic factors include gastrointestinal (non-colorectal) primary tumors [21–23]. In our study, 19% of patients had primary tumors arising from either the reproductive or genitourinary tracts (excluding adrenal), while 25% had foregut primary tumors (gastroesophageal, pancreas, duodenum, ampulla).

This work has identified a 5-year overall survival rate of 58%, in contrast to a rate of 36% published by Adam and colleagues in the largest such series to date [10]. This difference can be explained by the fact that the two largest groups of patients in the current series were also those with the best prognoses (breast cancer and melanoma, 21% of the cohort each). Although Adam et al. included 32% of breast cancer patients and 10% of melanoma patients [10], it is likely that they also chose to operate on many more patients than we did, perhaps including many who would not have been considered at our center. Finally, it is also important to consider that 85% of our patients had surgery after 2000, while Adam et al. included patients in 1983–2004 of which 41% were operated after 2000. Our patients are much more likely to have benefited from recent improvements in chemotherapeutic and biologic therapies, thus further improving our survival rate.

In this work, 21% of the primaries were melanoma, with 5-year overall survivals of 66% and 83% for ocular and other melanomas, respectively. Similarly, another small study has reported acceptable surgical treatment of melanoma liver metastases with reported prolonged survival rates: 3 year overall survival of 56% for primary ocular and 60% for cutaneous melanoma [24]. In contrast, work by Adam *et al* would suggest 5-year survivals of 21–22% [10]. Although surprising, the difference between our data and this large series is likely related to major differences in patient selection. It seems clear that the resection of liver metastases from melanoma warrants further study, particularly as it pertains to potential differences between ocular and cutaneous primaries, in the context of evolving biologic systemic therapies [25].

A large proportion of the patients in our study had metachronous disease (81%) with a long interval from primary diagnosis to metastasis of disease. In 2006, Earle *et al* showed that longer time interval between diagnosis of the primary tumors and diagnosis of liver metastases (disease free interval) was predictive of survival [23]. A number of other studies have agreed with these findings and also demonstrated increased survival from time of liver resection [10, 15, 16, 19, 26]. A prolonged interval without metastases following primary diagnosis is thought to be a surrogate marker for favorable tumor biology [21], and may partially account for the generally favorable outcomes obtained in this series.

There is growing evidence that hepatectomy offered to patients who have shown response to chemotherapy yields better results [3, 26]. In patients with large tumors, chemotherapy for downsizing can be considered [10]. The precise role of chemotherapy could not be established in the present study due to the disparate nature of the study group. It is well established that response to chemotherapy is associated with improved prognosis for CRLM, but that is not necessarily the case for NCNNNS lesions, as existing regimens vary widely based on the type of primary lesion. In the absence of effective chemotherapy regimens, the feasibility of resection is therefore a very important part of the pre-operative work-up. Other authors have shown that patients with more than one metastatic deposit, even if there is bilobar liver involvement, have a similar disease free survival as those with solitary lesions. This is dependent on R0 resectability of every lesion, as well as sufficient FLR [13].

There are several limitations of this retrospective, multicenter study. Our NCNNNS cohort is heterogeneous, being composed of patients with metastases from several different primary tumors. Breast and melanoma were well represented, but it is likely that their malignant behavior is not generalizable to less common primaries, particularly foregut carcinomas. Ideally, primary tumors would be divided based on their origin as well as their histology, but small numbers preclude this. Our small patient population also precluded analysis of possible prognostic variables. Finally, our patient population was highly selected, which likely contributed to our generally favorable outcomes.

This work adds to a growing body of literature pertaining to liver resection for NCNNNS metastases. At the current time, it is unlikely that a single institution will treat a sufficiently large number of patients with varying primary tumor entities to address this research topic definitely. As a result, institutional data should be pooled. We have presented a multi-center study showing that hepatic resection offered to carefully selected patients with NCNNNS metastases is an important component of the multidisciplinary management of these patients. Further large-scale multicenter pooling of institutional series is warranted, with the goal of establishing appropriate therapeutic algorithms for the management of resectable NCNNNS liver metastases.
